# Anti-EGFR mAb cetuximab therapy increases T cell receptor (TCR) diversity in the peripheral blood and focuses TCR richness in the tumor microenvironment

**DOI:** 10.1186/2051-1426-3-S2-P73

**Published:** 2015-11-04

**Authors:** Benjamin A Kansy, Yan Lin, Fei Ding, Sandra Poveda Gibson, Hyun-Bae Jie, Robert L Ferris

**Affiliations:** 1 Department of Otorhinolaryngology, University Hospital Essen, Germany; University of Pittsburgh Cancer Institute, Pittsburgh, PA, USA; 2 Biostatistics Facility, University of Pittsburgh Cancer Institute, Pittsburgh, PA, USA; 3 University of Pittsburgh Cancer Institute, Pittsburgh, PA, USA

## Introduction

T cell receptor (TCR) recognition of tumor antigen is essential for effective antitumor immunity. The immune system`s ability to respond to a broad spectrum of antigens requires a sophisticated selection of various TCR. So far, little is known about the role of TCR richness and clonality in the immune response to the EGFR-specific mAb, cetuximab. Therefore, we investigated differences in TCR sequences between HPV^+^ and HPV^-^ patients, as well as differences in sequence characteristics between T cells of peripheral blood mononuclear cells (PBMC) and tumor infiltrating lymphocytes (TIL). Additionally, we were able to investigate the TCR richness and clonality in samples pre- and post-treatment in a clinical single agent cetuximab trial.

## Material and methods

TCR genotyping was performed and quantified by Adaptive Technologies, Inc (Seattle, WA). Richness (S) was defined as total number of unique productive sequences. The Shannon Index is defined as H=-log∑p_i_(1-p_i_), p_i_ is the proportion of sequence *i* relative to the total sequences. The clonality is defined as 1-*H*/log(S). The maximum available number of samples from the same patient was 4, containing pre- and post-treatment PBMC and pre- and post-treatment TIL. Paired comparisons (pre vs. post) were done using the Wilcoxon Signed Rank tests. Comparisons between two independent groups (i.e. HPV^+^ vs. HPV^-^, or responders vs. non-responders) were accomplished with Wilcoxon Rank Sum tests.

## Results

Using a cohort of neoadjuvant, single-agent cetuximab treated HNSCC patients, 56 samples were analyzed for global TCR diversity. HPV^+^ and HPV^-^ patients did not significantly differ in clonality and richness pre- and post-treatment in either PBMC or TIL. However, cetuximab therapy significantly increased the richness of unique sequences in PBMC (p=0.0002). Most importantly, the responder group had a higher increase of richness post-treatment in PBMC. The difference reached a p-value of 0.03 post-treatment. On the contrary, this increase was not observed in TIL, which appeared to be more focused after cetuximab therapy.

## Discussion

TCR clonality and richness for PBMC and TIL seem to be independent from the patient's HPV status in HNSCC. Intriguingly, an increase in richness of TCR sequences in the periphery (PBMC) but a focusing in TIL was associated with an improved treatment response, suggesting an influence of peripheral quantity on intratumoral TCR quality.

